# Advances in Nanotechnology Research in Food Production, Nutrition, and Health

**DOI:** 10.3390/nu17152443

**Published:** 2025-07-26

**Authors:** Kangran Han, Haixia Yang, Daidi Fan, Jianjun Deng

**Affiliations:** 1 Shaanxi Key Laboratory of Degradable Biomedical Materials, Shaanxi R&D Center of Biomaterials and Fermentation Engineering, Biotech & Biomed Research Institute, School of Chemical Engineering, Northwest University, Xi’an 710069, China; 15635640698@163.com (K.H.); fandaidi@nwu.edu.cn (D.F.); 2 College of Food Science and Nutritional Engineering, China Agricultural University, Beijing 100083, China; hyang@cau.edu.cn; 3 State Key Laboratory of Vegetable Biobreeding, Institute of Vegetables and Flowers, Chinese Academy of Agricultural Sciences, Beijing 100081, China

**Keywords:** nanotechnology, food production, nutritional delivery, human health, safety

## Abstract

Nanotechnology, as a burgeoning interdisciplinary field, has a significant application potential in food nutrition and human health due to its distinctive structural characteristics and surface effects. This paper methodically examines the recent advancements in nanotechnology pertaining to food production, functional nutrition delivery, and health intervention. In food manufacturing, nanoparticles have markedly enhanced food safety and quality stability via technologies such as antimicrobial packaging, intelligent sensing, and processing optimization. Nutritional science has used nanocarrier-based delivery systems, like liposomes, nanoemulsions, and biopolymer particles, to make active substances easier for the body to access and target. Nanotechnology offers innovative approaches for chronic illness prevention and individualized treatment in health interventions by enabling accurate nutritional delivery and functional regulation. Nonetheless, the use of nanotechnology encounters hurdles, including safety evaluations and regulatory concerns that require additional investigation. Future research should concentrate on refining the preparation process of nanomaterials, conducting comprehensive examinations of their metabolic mechanisms within the human body, and enhancing pertinent safety standards to facilitate the sustainable advancement of nanotechnology in food production, nutrition, and health.

## 1. Introduction

**Figure 1:**
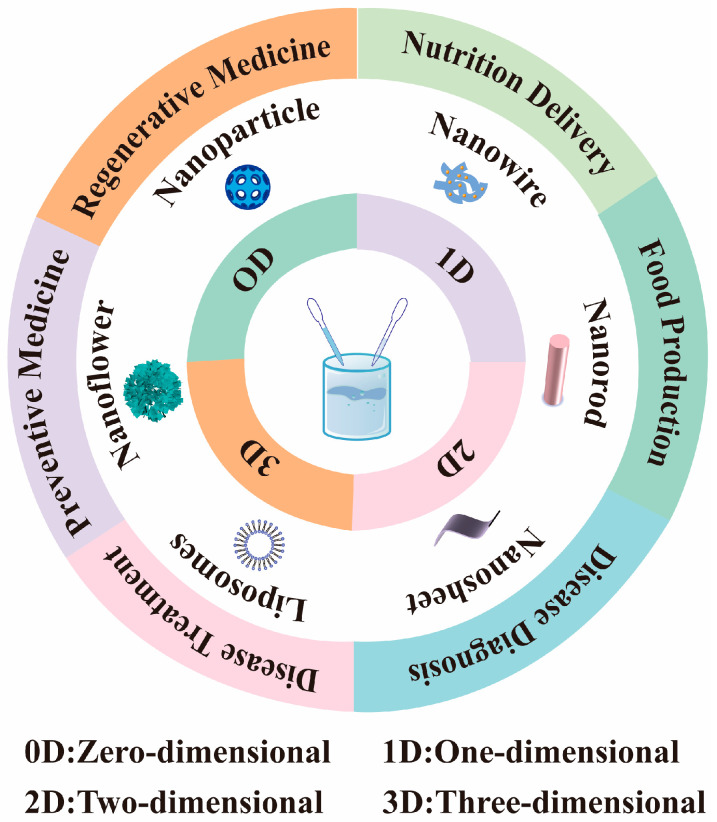
Nanomaterials for use in food production, nutrition, and health.

In recent years, global economic growth and rising living standards have heightened the public awareness of nutrition and health. Concurrently, advances in nanotechnology have created unprecedented opportunities in food science and nutritional health, offering innovative solutions to address global challenges in food safety, nutritional fortification, and health management. The origins of nanotechnology trace back to 1959 when Nobel Prize-winning physicist Richard Feynman first proposed the revolutionary concept that “there’s plenty of room at the bottom,” marking the embryonic stage of nanotechnology [1]. Modern nanotechnology involves the study, manipulation, and application of materials at the nanoscale [2]. Nanotechnology represents an interdisciplinary field spanning physics, chemistry, materials science, and biology. It achieves precise control over material properties, structures, and functionalities through the manipulation of individual atoms, molecules, or molecular clusters. Nanomaterials are formally defined as substances with at least one dimension confined to the nanoscale in three-dimensional space or materials composed of such nanoscale building blocks as their fundamental structural units [3,4]. Nanomaterials can be classified into inorganic nanomaterials and organic nanomaterials based on their composition. Inorganic nanomaterials are typically synthesized from metals, metal oxides, and silicon-based compounds. Organic nanomaterials primarily include polymer-based, lipid-based, carbon-based, protein, and peptide-derived nanostructures. Based on the differences in spatial dimensions, nanomaterials can be categorized into zero-dimensional (0D), one-dimensional (1D), two-dimensional (2D), and three-dimensional (3D) nanomaterials. These nanomaterials with different dimensions exhibit unique physical, chemical, and biological properties. They have shown great potential in food production, processing, packaging, nutritional fortification, and the development of functional foods. At the same time, they also provide new research directions for precision nutrition and personalized health management (Figure 1). This article provides a detailed introduction to the classification of nanomaterials and their applications in the fields of food science, nutrition, and health. It offers a theoretical basis for the research and development of nanomaterials in areas such as food production, functional nutrition delivery, and chronic disease management.

## 2. Classification of Nanomaterials

Nanomaterials can be classified into inorganic and organic categories according to their composition.

### 2.1. Inorganic Nanomaterials

Inorganic nanomaterials are nanoscale substances made from inorganic compounds, exhibiting distinct physical, chemical, and mechanical properties [5]. Inorganic nanomaterials are generally nanomaterials prepared from metals, metal oxides, and silicon-based materials. These nanomaterials can achieve a variety of shapes through controlled synthesis methods, such as nanoparticles, nanowires, nanorods, nanofilms, nanoporous materials, and nanoflowers. In particular, nanosheets and nanofilms, as emerging types of nanomaterials, have attracted significant attention from the scientific community in recent years due to their extraordinary properties that are different from those of nanoparticles and bulk nanomaterials. For example, black phosphorus (BP) nanosheets, which are the most extensively studied, have garnered considerable interest [6]. BP nanosheets demonstrate potential as viable inorganic nanosystems for therapeutic use owing to their intrinsic biodegradability and distinctive monophosphorus structure. The therapeutic antidepressant fluoxetine was effectively adsorbed onto the surface of BP nanosheets through electrostatic interactions. The study’s results indicated that this loading method utilizing near-infrared laser irradiation greatly reduced the treatment duration for depression and effectively mitigated the negative effects associated with conventional medicine [7]. Diverse geometries provide distinct physical and chemical properties to materials, along with varied applications (Table 1).

### 2.2. Organic Nanomaterials

Organic nanomaterials are functional substances composed of carbon-based molecules (e.g., polymers, lipids, proteins, tiny organic compounds, etc.) ranging in size from 1 to 1000 nanometers, exhibiting distinctive physicochemical features [23]. Organic nanomaterials typically encompass nanomaterials derived from polymers, liposomes, carbon-based substances, proteins, and peptides. Although inorganic nanomaterials are widely used in the fields of medicine and food, organic nanomaterials have unique advantages in these areas due to their inherent biocompatibility, biodegradability, and functional diversity. Future research will focus on precision medicine, green synthesis, and industrial applications.

#### 2.2.1. Polymer Nanomaterials

Polymeric nanomaterials are useful materials at the nanoscale made from natural or synthetic polymers, characterized by adjustable physicochemical qualities and superior biocompatibility [24]. These have emerged as a fundamental research focus in nanotechnology owing to their substantial drug-loading capacity, targeting capabilities, sustained release characteristics, and responsiveness to stimuli, alongside their extensive applications across diverse domains, including drug delivery systems, biosensors, catalysts, nanocomposites, agriculture, and environmental science [25].

The utilized polymeric materials may be either synthetic or natural. Polyvinyl alcohol (PVA), polyethylene glycol (PEG), polycaprolactone (PCL), amphoteric polymers, and poly (lactic acid)–hydroxyglycolic acid copolymers (PLGA) are some of the polymer monomers that are commonly used in synthetic polymer nanomaterials. These monomers allow for precise structural modification through molecular designs and are extensively employed in biomedical applications owing to their superior biocompatibility. Amphiphilic ionic polymers comprise cationic and anionic groups within the molecular structure. They possess elevated dipole moments and significantly charged groups; however, they are often electrically neutral [26].

In contrast to synthetic polymers, natural polymers, including chitosan, hyaluronic acid, alginate, and cellulose, exhibit excellent biocompatibility and biodegradability, are predominantly sourced from nature, and are extensively present in plants, animals, and microbes [27]. Alginate and chitosan are often utilized substances in the fabrication of nanoparticles. Alginate, an unbranched anionic polysaccharide, possesses many carboxyl groups, rendering it pH-responsive and enhancing the bioavailability of lipophilic actives [28]. Chitosan (CS), the sole natural cationic polysaccharide, is extensively utilized in medical and pharmaceutical applications owing to its superior biocompatibility, biodegradability, and minimal immunogenicity [29]. In recent years, chitosan has undergone significant modification using hydrophilic or hydrophobic agents to produce amphiphilic chitosan derivatives [30]. Table 2 lists the common components of polymer nanomaterials and their advantages.

#### 2.2.2. Liposomes

Liposomes are very small bubble-like structures made of phospholipids that have a two-layer structure similar to biological membranes. They comprise a phospholipid bilayer, an aqueous core that encapsulates water-soluble drugs, and a lipid layer containing lipid-soluble molecules [41]. Various categories of active molecules, including vitamins, enzymes, antimicrobial peptides, essential oils, phenolic compounds, antioxidants, food additives, flavors, fatty acids, pharmaceuticals, proteins (peptides), antigens, and nucleotides, can be encapsulated within liposomes. Liposomes encapsulate both hydrophobic and hydrophilic substances, preventing the combinatorial degradation of the encapsulation and facilitating targeted release at specified sites [42,43,44]. Although liposomes have diverse applications in medicine administration, cosmetics, and the food industry, they exhibit several limitations, including unstable physicochemical qualities, a vulnerability to acids, bases, salts, light, heat, and oxidizing agents, as well as potential immunogenicity hazards. Consequently, contemporary researchers are exploring methods to enhance the stability of liposomes and diminish their immunogenicity, including the incorporation of antioxidants (vitamin E), glycoprotectors (alginate, chitosan, sucrose, etc.), or alterations to surface polymers. Guan et al. developed a chondroitin–chitosan bilayer-modified nanoliposome for encapsulating the water-soluble pigment betalains. In vitro digestion experiments demonstrated that this bilayer-modified nanoliposome displayed enhanced stability and a slower release of betalains, resulting in a superior bioavailability compared to both the pure nanoliposome and the chitosan-modified nanoliposome [45].

#### 2.2.3. Solid Lipid Nanoparticles

Solid lipid nanoparticles consist of a solid lipid matrix and typically range in size from 50 to 300 nm, offering an innovative method for encapsulating lipophilic bioactives [46]. Solid lipid nanoparticles integrate the benefits of liposomes and polymer nanoparticles, are often composed of solid materials (e.g., triglycerides) at an ambient temperature, and exhibit superior physical stability compared to liposomes. Biodegradable and highly biocompatible natural lipid components, such as stearic acid and beeswax, are typically employed. We can enhance the bioavailability and stability of fat-soluble bioactive compounds by employing solid lipid nanoparticles for their distribution. Controlled the drug release from solid lipid nanoparticles (SLNs) was accomplished by modifying the lipid composition and implementing surface alterations to improve targeted delivery and boost stability [47]. Lin et al. used the film dispersion method to integrate lemon eucalyptus essential oil inside SLNs. They were able to achieve a maximum encapsulation rate of 22.47% in the liposomes. Investigations into the release rate and storage stability of lemon eucalyptus essential oil demonstrated that the oil contained in solid liposomes exhibited a remarkable stability [48]. Notwithstanding the significant benefits of solid lipid nanoparticles in the delivery of lipid-soluble bioactive compounds—such as enhanced encapsulation efficiency, drug loading, bioavailability, and scalability—limitations persist in their practical uses. During preparation and storage, the solid lipids in SLNs crystallize, resulting in the formation of a stiff core. The crystalline morphology of lipids evolves over time; for instance, the transition from the α-crystalline to the β-crystalline can impede the mobility of drug molecules, resulting in the spatial confinement of the drug and potentially causing its extrusion from nanoparticles, thereby diminishing the drug’s encapsulation efficiency [49]. SLNs typically exhibit an initial burst release, wherein the drug is rapidly discharged at the onset of administration, potentially compromising the controlled release and hindering the intended continuous release of the drug.

#### 2.2.4. Nanoemulsions

Nanoemulsions are transparent or semi-transparent dispersed systems consisting of oil, water, a surfactant, and a co-surfactant, with particle sizes typically between 20 and 200 nm [50]. They offer advantages such as kinetic stability and high bioavailability, comparable to aqueous-phase viscosity, making them suitable for injections or transdermal administration. Consequently, they are extensively utilized in pharmaceuticals, cosmetics, and food products. Nanoemulsions are often classified as oil-in-water (O/W), water-in-oil (W/O), and bicontinuous (oil–water interpenetration) based on the characteristics of the bioactive molecule intended for encapsulation [51]. The choice of emulsifying agents and their respective ratios is essential for producing a stable emulsion system with the appropriate particle size. The components of the oil phase comprise fatty acids, vegetable oils, mineral oils, and fish oils [52], and the selection of the oil phase type influences the content and bioavailability of bioactive compounds. The aqueous phase may consist of pure water, salt, a phosphate buffer, or an aqueous solution containing a designated medicinal component, selected based on the application needs of the nanoemulsion to improve its functionality and stability. Nanoemulsion preparation techniques can be categorized into low-energy emulsification and high-energy shear approaches. Generally, the oil and emulsifier phases are combined, bioactive molecules are incorporated, and the mixture is agitated vigorously until dissolved, followed by the addition of the aqueous phase. Subsequently, intense forces are produced by high-pressure homogenizers, microjets, and ultrasonic devices, which fragment the oil and aqueous phases into nanometer-sized droplets [53,54]. The high-energy shear approach can produce nanoemulsions with small particle sizes and a uniform dispersion rapidly; nevertheless, it necessitates a significant energy input and is susceptible to elevated temperatures. Conversely, the low-energy emulsification technique establishes the proportions of the oil phase, emulsifier, co-emulsifier, and medicine based on its ternary phase diagram; thoroughly combines the components; and gradually incorporates the aqueous phase while stirring to produce a transparent or translucent nanoemulsion [55]. The process does not necessitate complex equipment or the introduction of substantial mechanical energy and operates under mild reaction conditions suitable for temperature-sensitive or unstable bioactive compounds.

#### 2.2.5. Carbon-Based Nanomaterials

Carbon-based nanomaterials are nanoscale substances comprising the element carbon, encompassing carbon nanotubes, graphene, carbon dots, and more nanomaterials. The extensive array of industrial and medical applications of carbon nanomaterials is attributable to the distinctive chemical characteristics of carbon and the variety of potential carbon nanostructures. The variety of carbon nanostructures and their structural and chemical characteristics arise from differences in sp, sp2, and sp3 hybridization [56]. Carbon-based nanomaterials exhibiting excellent biocompatibility, an extensive specific surface area, and distinctive optical properties can effectively encapsulate therapeutic molecules for targeted delivery and sustained release, as well as for biomedical imaging and diagnostics. Furthermore, several carbon nanostructures include antibacterial and antiviral characteristics that may be utilized to create novel coatings for medical equipment or food packaging, hence prolonging the food shelf life. The utilization of carbon-based nanomaterials in pharmaceuticals, cosmetics, and food applications reveals significant promise; however, it is imperative to focus on their safety and risk evaluation to guarantee their reliability and sustainability in practical use.

#### 2.2.6. Protein/Peptide Nanomaterials

Protein/peptide nanomaterials are nanoscale entities composed of protein or peptide molecules by processes such as self-assembly and cross-linking, often ranging from 1 to 100 nm in size. Their shapes are varied, manifesting as nanoparticles, nanofibers, nanotubes, and other forms [57]. There are three primary methods for their preparation: (1) The self-assembly method employs non-covalent interactions (e.g., hydrogen bonding, hydrophobic contacts, electrostatic interactions, etc.) among protein or polypeptide molecules to induce the spontaneous formation of structured nanostructures [58]. (2) The chemical cross-linking approach involves the use of chemical reagents to join protein or peptide molecules, resulting in the formation of stable nanostructures [59]. (3) Physical techniques, encompassing ultrasound, high-pressure homogenization, spray drying, etc., physically distribute proteins or peptides into nanoscale particles [60]. Protein/peptide nanomaterials possess good biocompatibility, biodegradability, and cell adhesiveness. They can serve as drug carriers to enhance the stability and solubility of drugs. Additionally, they can be used to construct tissue engineering scaffolds, which promote cell adhesion, proliferation, and differentiation. They can also function as recognition elements in biosensors for the detection of biomarkers, pathogens, and other targets, with a high sensitivity and specificity. Moreover, many protein/peptide nanostructures possess antibacterial capabilities and can serve as food preservatives to prolong the shelf life of food.

## 3. Nanotechnology in Food Production, Nutrition, and Health

### 3.1. Application of Nanotechnology in Food Production

Nanotechnology has introduced transformative advancements in food and nutrition via novel materials and methodologies. The food production process encompasses the entire industry chain, from raw material processing to final products. Its primary process units consist of essential processing operations, product preservation, packaging technologies, logistics and transportation, and warehouse management [61]. The primary functional objectives of these processes are the inactivation of harmful microorganisms and enzyme systems in food products via physical or chemical methods, the effective removal or degradation of potentially toxic constituents, and the supplementation of essential nutrients through nutritional fortification techniques to augment the nutritional value of products [62]. In food processing and preservation, nanotechnology can be employed to create innovative food additives and ingredients that enhance the texture, flavor, color, and stability of food. Smart packaging employs nanosensors and active antimicrobial coatings for the real-time monitoring of food safety and the prolongation of shelf life. In nutrient delivery, nanocarriers (such as lipid nanoparticles and protein nanoparticles) facilitate a tailored release to enhance the nutrient absorption efficiency and minimize negative effects. Moreover, nanotechnology facilitates the advancement of personalized nutrition tailored to the health requirements of diverse populations via sophisticated detection and delivery systems.

#### 3.1.1. Application of Nanotechnology in Food Processing

Food processing encompasses varied production methods that require distinct raw material attributes and finished product specifications, hence offering extensive opportunities for new uses of nanotechnology. The implementation of nanoscale interventions can systematically optimize and enhance several critical operations within the current processing system, markedly enhancing the efficiency and quality control of conventional food processing. Nanotechnology’s application in food processing can be classified into two primary types based on its mechanism of action: direct application and indirect application. Nanomaterials are used as functional ingredients in food systems through direct application. These nanomaterials include nano-preservatives, nano-antimicrobial agents, nanoemulsifiers, antioxidants, colorants, and bioactive molecules, like nanosized starch and nanozinc oxide. These materials can enhance the texture and mouthfeel of food products, extend their shelf life, augment nutrient bioavailability, improve the stability of food emulsions, and inhibit oil–water separation [62,63,64,65]. Indirect application denotes the utilization of nanotechnology in equipment during food processing, as opposed to direct interactions with food [66]. Table 3 demonstrates the applications of nanotechnology in food manufacturing and processing.

#### 3.1.2. Application of Nanotechnology in Food Packaging

In the food industry production chain, packaging serves a vital protective role. Packaging materials, as a crucial element of the product processing chain, efficiently mitigate the influence of diverse external influences on food quality through several barriers [77,78,79]. Food packaging can mitigate the impacts of temperature variations and humidity changes on products by controlling the physical properties of the packaging material. Furthermore, the implementation of specialized barrier technologies can avert oxidation and degradation resulting from the infiltration of environmental gases, such as oxygen. Additionally, the packaging system can prevent microbial contamination, while also possessing anti-leakage and anti-physical shock properties. However, traditional food packaging materials have obvious shortcomings in terms of functionality. These materials generally have significant weaknesses in terms of barrier properties, particularly a low barrier efficiency against gases such as oxygen and water vapor. They lack active preservation capabilities, allowing microorganisms to grow prematurely and accelerating food spoilage [80]. The expanding capabilities of nanostructures are furthering the application of nanotechnology in food packaging [81]. Nanotechnology preserves the freshness and quality of food by enhancing the mechanical qualities, barrier properties, and thermal stability of packaging materials to inhibit the ingress of oxygen, moisture, and aromas [82]. Table 4 enumerates various nanomaterials and their applications for enhancing food packaging and thus prolonging shelf life.

#### 3.1.3. Application of Nanotechnology in Food Testing

In the context of the global socio-economic advancement and ongoing population growth, the assurance of food quality and safety has emerged as a significant strategic concern for the international community. Additionally, the overall increase in public health awareness has led various sectors of society to demand elevated food safety standards. Food safety governance faces multiple challenges, including key risk factors such as chemical contaminant residues during agricultural production; microbial growth during food processing, storage, and transportation; and contamination by naturally occurring biotoxic substances [105,106]. In reaction to these significant safety risks, the advanced evolution of contemporary analytical testing and detection technologies has offered crucial technical assistance for food safety surveillance. Rapid detection methods and real-time monitoring technologies are becoming increasingly vital in the food safety risk monitoring system due to their technical advantages, including the ease of operation and swift reactions. Traditional chromatographic and spectroscopic techniques are constrained in large-scale applications due to their instrumentation and cost; conversely [107,108,109], nanotechnology plays a pivotal role in food testing and monitoring owing to its affordability, portability, and superior detection efficiency, thereby transforming food safety and security [110,111,112,113].

Nanosensors represent a significant application of nanotechnology in food testing, enabling the shrinking of detection units that are intelligently integrated into packaging systems, thus facilitating a transformative shift from “post-sampling” to “comprehensive process monitoring [114].” Aflatoxin B1 (AFB1), the most hazardous variant of the mycotoxin family, is frequently found in diverse crops and their processed derivatives [115]. AFB1 was designated as a Group 1 carcinogen by the International Agency for Research on Cancer (IARC) in early 2002, owing to its mutagenic, teratogenic, and carcinogenic properties [116,117]. Consequently, the Food and Agriculture Organization of the United Nations (FAO) has established maximum permissible limits (MPLs) for AFB1 in food products. To comply with food safety standards and reduce labor costs and trade losses in regulation, researchers are persistently seeking rapid, portable, and highly sensitive technologies for AFB1 quantification. Dai et al. devised an innovative multimodal (photothermal/colorimetric/fluorescent) nano-enzyme-linked immunosorbent assay that employed magnetic nanoparticles (MNPs) as carriers, facilitating the in situ growth of Prussian blue nanoparticles (PBNPs) on their surfaces in an acidic milieu, thereby successfully fabricating an MNPs@PBNPs composite probe. The integration of fluorescence detection and photothermal response signals resulted in the enhanced sensitivity of the detection method; additionally, the colorimetric signal was markedly improved through the photothermal effect under near-infrared (NIR) excitation and the catalytic activity of the peroxidase-like enzyme (POD) [118].

### 3.2. Application of Nanotechnology in Functional Food Delivery

The incidence of chronic non-communicable diseases in modern society is growing significantly. Reports indicate a rising prevalence of chronic diseases, including diabetes, hypertension, obesity, coronary heart disease, and chronic bronchitis, largely attributable to lifestyle factors such as inadequate food habits and insufficient physical activity. Incorporating functional components into foods and dietary supplements is an effective approach for preventing and alleviating chronic diseases [119,120].

#### 3.2.1. Application of Nanotechnology as Functional Ingredient Delivery System

**Figure 2:**
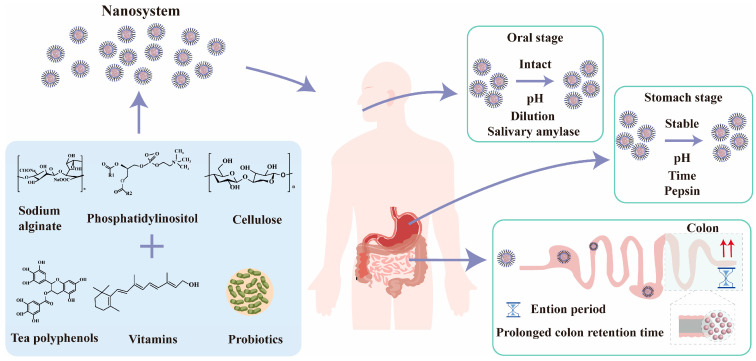
A schematic representation of the digestion and absorption of polymeric nanoparticles infused with functional food constituents throughout the gastrointestinal tract.

Functional components offer substantial benefits in nutritional supplementation, chronic disease prevention, immune enhancement, digestive function improvement, and metabolic health promotion, thereby addressing the health requirements of diverse populations and boosting overall well-being [121]. Nonetheless, these functional substances encounter numerous bioavailability obstacles from their intake to their absorption and usage by the body. The primary constraints encompass environmental stability concerns (oxygen, light, temperature), the inadequate dispersion resulting from low water solubility, the susceptibility to pH fluctuations, and the vulnerability to enzyme breakdown within the gastrointestinal system [122,123,124]. In recent years, researchers have increasingly focused on nanotechnology for bioactive substance delivery systems, utilizing proteins, polysaccharides, and other polymers through molecular designs and structural regulation. This approach effectively enhances the stability and bioavailability of embedded active ingredients, facilitating the efficient loading and controlled release of various functional components. Nanoparticles can be engineered to deliver functional ingredients to the target site by employing various release mechanisms, contingent upon the reaction conditions. As shown in Figure 2, pH-responsive nanocarriers exhibit reversible conformational changes in their molecular structure in response to varying pH levels across different segments of the gastrointestinal tract. In the highly acidic milieu of the stomach (pH 1.0–3.0), such substances can preserve a stable structure and efficiently shield the encapsulated nutritional active components from destruction. Upon entering the neutral milieu of the small intestine, the nanoparticles disaggregate to facilitate the regulated release of the active compound [125]. This tailored release feature enhances both the delivery efficiency of the bioactive molecule and its absorption and utilization in the digestive tract.

Recent research has illustrated the significance of nanotechnology in functional foods. Polyphenolic compounds are a category of secondary metabolites prevalent in plant-based diets, known for their superior antioxidant, anti-inflammatory, and cardiovascular protective properties, and are extensively utilized in functional additives [126]. Nonetheless, its bioavailability is constrained by its inadequate solubility and stability [127]. Curcumin (CUR) is a prevalent polyphenol recognized for its biological actions, including anti-inflammatory, anticancer, antibacterial, antiparasitic, and antioxidant properties. Zhang et al. formulated oleanolic acid-coated curcumin nanoparticles (OC NPs), thereby augmenting the anti-inflammatory and antioxidant properties of curcumin and facilitating stomach mucosal healing. The OC NPs were spherical in shape with an average diameter of 192.6 ± 1.26 nm. They exhibited a CUR loading capacity of 89.35 ± 0.63%, demonstrating excellent solubility and stability and a high drug encapsulation efficiency. These NPs maintained their structural integrity within biological systems and showed an outstanding biocompatibility. In vivo, OC NPs exhibited a prolonged retention in the stomach, facilitated a targeted drug delivery system, and significantly mitigated ethanol-induced gastric ulcers in mice by reducing inflammatory cytokines and enhancing antioxidant levels [128].

#### 3.2.2. Application of Nanotechnology as Probiotic Delivery System

Intestinal flora are intricate microbial communities residing in the human digestive tract, serving a varied regulatory function in sustaining the host physiological homeostasis [129]. Disturbances in the dynamic equilibrium of this system, caused by intrinsic factors (such as genetic predisposition) or extrinsic factors (including dietary composition, antibiotic administration, and pathogenic microbial infections), may precipitate an imbalance in the microbial community structure, potentially resulting in various digestive disorders, metabolic irregularities, and neurological dysfunctions [130]. Studies indicate that live probiotics can modulate the gut flora; nevertheless, the issue lies in ensuring their survival against physical and chemical assaults during intake and subsequent gastrointestinal transit. Hosseini et al. employed nanoencapsulation to enhance the gastrointestinal tolerance of probiotics. The lecithin solution was uniformly blended with the probiotic suspension using the vortex mixing method to create nanoliposomes encapsulating probiotics, and the stability was enhanced by applying a polysaccharide–protein bilayer protective film on the liposome surface through electrostatic adsorption. The outcomes of in vitro simulated digestion trials indicated that the polysaccharide–protein composite layer effectively safeguarded the encapsulated probiotics from the detrimental effects of the gastric acid and digestive enzymes by improving the stability of the nanocarriers [131].

### 3.3. Nanotechnology in Human Health

Due to alterations in nutrition, lifestyles, and environments, an increasing number of individuals are experiencing chronic diseases, including diabetes, hypertension, obesity, cancer, and chronic inflammation. Chronic diseases adversely impact the human body in several ways, compromising both the physical health and the quality of life of patients, while also imposing a significant burden on families and society. Consequently, bolstering the prevention and management of chronic diseases, together with augmenting public health awareness and self-management skills, is crucial for improving the prognosis of chronic disease patients and alleviating the burden on families and society. Nanotechnology, as an advanced discipline, is significantly transforming the maintenance and treatment of human health. It presents unparalleled prospects in disease diagnosis, therapy, prevention, and regenerative medicine via the manipulation of matter at the nanoscale.

#### 3.3.1. Nanotechnology in Disease Diagnosis

The early detection of disorders and the monitoring of physical conditions are essential for patient treatment and recovery. A precise diagnosis enables physicians to swiftly identify the etiology and status of the ailment, thus facilitating the development of the most appropriate treatment strategy for the patient, enhancing both the cure rate and the recovery speed. Early detection can prevent disease progression, diminish complications, markedly enhance the prognosis and quality of life for patients, and alleviate their suffering and financial burden. Consequently, the advancement of portable and cost-effective diagnostic tools facilitating a real-time and on-site diagnosis has garnered significant interest [132]. The utilization of nanotechnology in disease diagnosis has emerged as a pivotal advancement in contemporary medicine, markedly enhancing the sensitivity, accuracy, and speed of diagnostics through the distinctive properties of nanomaterials, thereby offering substantial support for the early detection and targeted treatment of diseases [133]. Nanotechnology is mostly utilized in imaging technologies, biosensors, portable detection devices, skin interface devices, real-time detection, and dynamic imaging.

Nano-imaging technology employs nanoparticles as contrast agents or probes to markedly enhance the resolution and sensitivity of medical imaging [134]. Biomedical imaging technology holds indispensable significance in contemporary disease diagnosis and therapy, particularly in the clinical management of malignant tumors and other critical illnesses. This technology enables the dynamic monitoring of lesion growth by non-invasive imaging methods, offering essential assistance for precision medicine. These real-time, intuitive imaging capabilities enhance diagnostic and therapeutic targeting and, crucially, offer an objective foundation for creating personalized treatment plans, thereby maximizing the overall therapy efficacy [135]. The three primary imaging modalities presently available are fluorescence imaging (FI), magnetic resonance imaging (MRI), and computed tomography (CT). Indocyanine Green (ICG) is presently sanctioned by the U.S. Food and Drug Administration (FDA) as an organic near-infrared fluorescent dye for medical diagnostic applications [136]. Chen et al. selected MIL-100 (Fe) as the carrier material to facilitate the efficient loading of ICG due to its distinctive porous structure. To improve tumor targeting, hyaluronic acid (HA) was chemically coupled to the surface of pre-assembled nMOF, resulting in the formation of MOF@HA@ICG composite nanoparticles with active targeting capabilities. The nanoparticles were effectively utilized as fluorescence enhancers in tumor imaging owing to their increased accumulation of ICG in tumors and their heightened imaging intensity. Moreover, fluorescence imaging demonstrated that Fe-MOF decreased the ICG breakdown and leakage in vivo prior to arriving at a specific and targeted lesion site inside the tissue [137].

**Figure 3:**
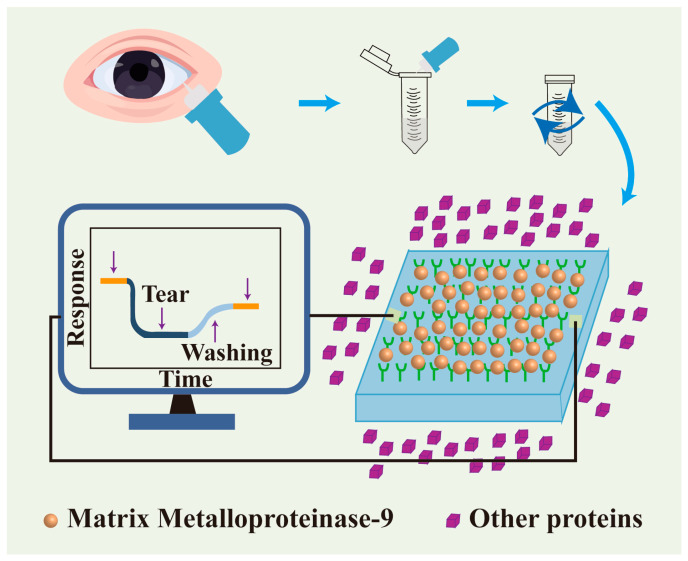
The utilization of nanotechnology in disease diagnosis.

Biosensors are primarily designed utilizing the high specific surface area and surface functionalization characteristics of nanomaterials. They can accurately identify disease-associated biomarkers, such as particular proteins or nucleic acids, in serum. Consequently, these sensors facilitate the early diagnosis of illnesses [138,139]. Matrix metalloproteinase-9 (MMP-9) is a protease released by the corneal epithelium; increased expression levels compromise the barrier function of the corneal epithelium, and the concentration of MMP-9 in tear fluid corresponds with the clinical manifestations of dry eye [140]. Therefore, as shown in Figure 3, Lu et al. developed a novel biosensor using silicon nanowire field-effect transistors (SiNW FETs) to measure the concentration of MMP-9 in human tears. They used a modified controllable process to make the SiNW dimensions more uniform and to keep their performance stable by optically calibrating it at low salt concentrations. Using this method, they were able to accurately measure the amount of MMP-9 in both normal and human tears. The results showed a strong link to the results of an enzyme-linked immunosorbent assay (ELISA). Furthermore, the quantities of the tear MMP-9 assessed by this method were associated with treatment outcomes in patients suffering from dry eye [141].

#### 3.3.2. Nanotechnology in Disease Treatment

**Figure 4:**
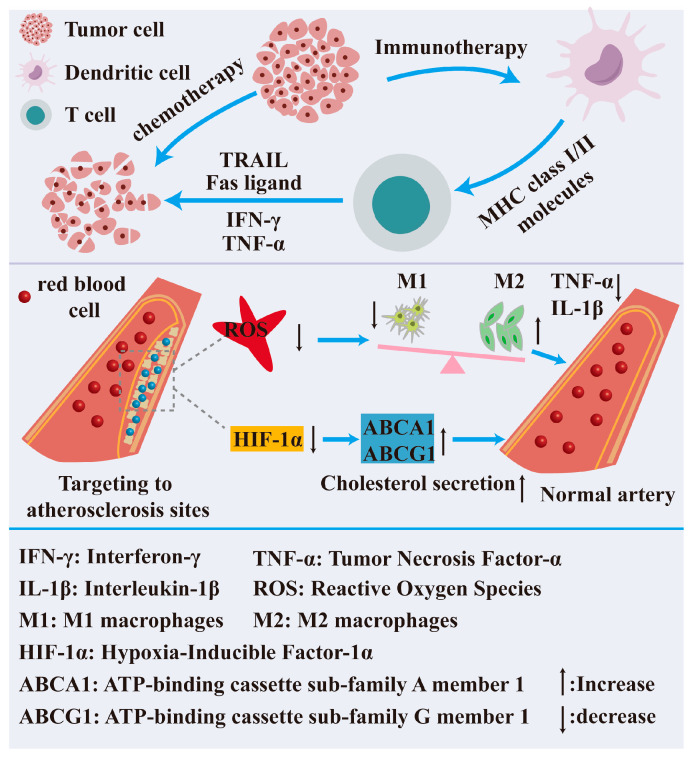
The utilization of nanotechnology in disease treatment.

The worldwide disease spectrum is experiencing substantial alterations, with the epidemiological attributes of numerous diseases exhibiting ongoing deterioration. This serious public health crisis presents new difficulties to contemporary medical treatment systems, necessitating the development of more targeted and efficient therapeutic procedures. The application of nanotechnology in disease treatment has become a major focus of modern medicine. By leveraging the unique properties of nanomaterials, it significantly enhances the precision, efficacy, and safety of therapies, showing great promise in chronic disease management (Figure 4). In particular, nanotechnology holds substantial potential for treating cancer and cardiovascular diseases.

Cancer is a multifaceted disease defined by uncontrolled, rapid cellular proliferation, potentially leading to the development of tumors [142]. Cancer treatment typically necessitates a multifaceted approach, incorporating surgery, radiation, chemotherapy, targeted therapy, and immunotherapy. Presently, radiation therapy, surgery, and chemotherapy constitute the primary therapeutic modalities for cancer; nevertheless, these approaches may inflict damage on healthy cells and result in various adverse effects. In recent years, the ongoing advancement of nanotechnology has prompted researchers to explore its use in cancer treatment. Nanotechnology has demonstrated significant potential and creativity in cancer treatment, offering renewed hope for addressing this worldwide medical crisis. Nanotechnology leverages the distinctive physical, chemical, and biological features of nanoparticles to achieve accurate drug delivery, targeted therapy, multimodal therapy, and real-time monitoring, thereby enhancing the efficacy and safety of cancer treatment. Chang et al. introduced a multifunctional nanoplatform utilizing Ti_3_C_2_-MXene Au nanocomposites to accomplish the integration of photothermal therapy, enzymatic kinetic therapy, and anti-tumor immunotherapy, supplemented by dual-mode in vivo imaging through photoacoustic and thermal methods [143]. Lan et al. created a light-responsive multifunctional nanosystem for cancer therapy. The BP nanosheets raised temperatures and damaged the mitochondria through their photothermal and photodynamic properties. This made the immune system’s ability to kill tumor cells stronger through immunogenic cell death (ICD). Concurrently, the intra-tumor administration of decitabine produces G2/M cell cycle arrest, hence enhancing the death of tumor cells and facilitating synergistic photothermal and photodynamic therapy in cancer treatment [144]. Zhong et al. created a Ga/Zn diatomic nanoconjugate enzyme (Ga/Zn-NC) that works like both peroxidase and glutathione oxidase but has different catalytic properties. This design creates a catalytic cascade that causes oxidative damage and makes breast cancer cells more sensitive to ferroptosis. At the same time, it releases gallium that has “pseudo-iron” properties that stop iron metabolism and start the self-amplifying ferroptosis pathway, which has a therapeutic effect on tumors. The release of gallium exhibiting “pseudo-iron” activity interferes with the iron metabolism and triggers the self-amplifying iron death pathway, leading to a notable therapeutic impact on malignancies [145].

Cardiovascular diseases (CVDs) have emerged as a significant and escalating public health issue worldwide, representing one of the most substantial health burdens and resulting in the highest incidence of disability and fatalities annually [146]. These diseases frequently exhibit analogous risk factors, encompassing metabolic irregularities (such as lipid and glucose metabolism disorders), circulatory dysfunction (e.g., chronic hypertension), and redox imbalances, which collectively disrupt the normal operation of various biological processes within the body [147,148]. Oxidative stress significantly contributes to the onset and advancement of cardiovascular disease [149]. Excessive free radicals assault proteins, lipids, and DNA within cells and tissues, impairing their function, obstructing normal signaling pathways, and inflicting damage on the body [150]. Nano-enzymes provide distinct benefits compared to genuine enzymes and traditional enzyme mimics, such as a reduced cost, easier manufacturing, and enhanced stability [151]. Atherosclerosis (AS) is a common and severe cardiovascular condition that may result in various irreversible consequences [152], such as myocardial infarction and heart failure, significantly endangering patients’ health and lives [153]. Consequently, An et al. formulated a synergistic amalgamation of endogenous H_2_S gas therapy with a polymerase-like nano-enzyme, designated LyP−1Lip@HS, for the treatment of AS. Low-intensity focused ultrasound (LIFU)-produced cavitation disturbed the lipid membrane LyP−1Lip@HS, thereby activating the enzyme-like activity of hollow mesoporous Prussian blue (HMPB) and facilitating the release of the endogenous H_2_S donor S-allyl-L-cysteine (SAC). LyP-1Lip@HS offers a targeted and regulated therapeutic strategy to mitigate oxidative stress, inflammation, and lipid metabolism abnormalities, positioning it as a potential treatment for AS [154]. Nano-enzymes serve as antioxidants and can function as medication transporters without compromising their catalytic activity. Chen and others created a nanomedicine called Col@HMnO2-MM that is loaded with colchicine and has hollow mesoporous manganese dioxide. This medicine can avoid being phagocytosed by immune cells, improves the blood flow in living things, targets inflammatory sites in atherosclerosis to release drugs more effectively, and reduces inflammation by getting rid of reactive oxygen species. In vivo tests indicated that the nanomedicine markedly enhanced medication efficacy and diminished inflammatory factor levels, lipid accumulation, and plaque formation without notable long-term harm [155].

#### 3.3.3. Nanotechnology in Regenerative Medicine

**Figure 5:**
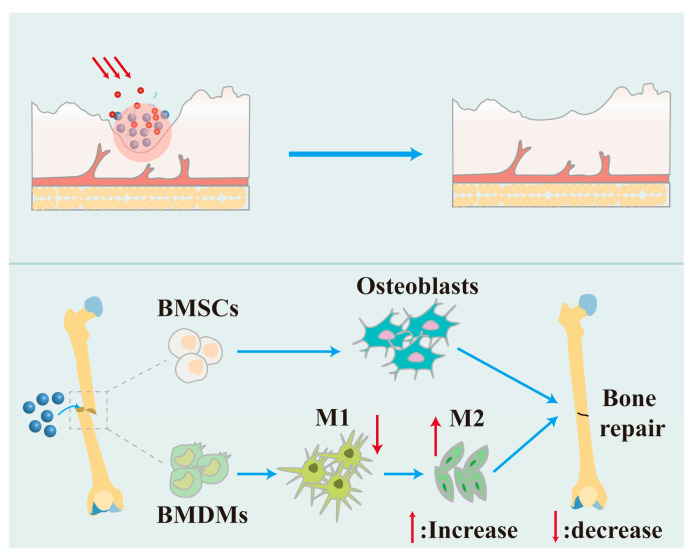
The utilization of nanotechnology in regenerative medicine.

The distinctive physical and chemical characteristics of nanomaterials allow them to replicate the microenvironment found in living creatures, creating optimal circumstances for cellular proliferation, differentiation, and tissue regeneration. Nanotechnology has emerged as a pivotal force in advancing regenerative medicine, offering unprecedented opportunities for tissue repair and regeneration. It holds significant clinical translation potential, particularly in biomedical applications such as wound healing and bone tissue regeneration (Figure 5).

The skin, being the organ system with the greatest surface area in the human body, executes numerous essential physiological activities for the preservation of bodily homeostasis [156]. The organ, beyond its fundamental protective function, participates in other physiological regulating processes, including environmental stimuli sensing, thermoregulation, and the maintenance of the water–electrolyte balance. Clinically, the integrity of skin tissue can be compromised by several endogenous and exogenous variables, such as trauma, burns, surgical procedures, and consequences from metabolic diseases [157]. During wound healing, things like an infection, a persistent inflammatory response, and problems with the blood vessels or nerves can stop the healing process from moving forward. This keeps the wound in the inflammatory or proliferative phase. This hinders the transition to the maturation remodeling phase, significantly disrupting the normal tissue repair process and resulting in the formation of a chronic wound [158]. Owing to significant advancements in nanotechnology in recent decades, several nanomaterials have been utilized in wound healing. Certain nanomaterials possess multifunctional properties, including antioxidant, pro-angiogenic, and antimicrobial capabilities, which can efficiently modulate the local microenvironment of wounds, manage infections, and facilitate the neovascularization and re-epithelialization of the skin, thereby expediting the wound healing process. Hong et al. formulated a nanospray utilizing polyacrylic acid (PAA)-cross-linked amorphous calcium peroxide (CaO_2_) nanoparticles for the management of infected wounds. In vitro and in vivo studies indicated that the nanoparticles facilitated wound healing by inhibiting bacterial survival, enhancing angiogenesis, and increasing cell proliferation and migration, among other capabilities [159]. Liu et al. engineered a copper-based nano-enzyme with a spectrum reactive oxygen species (ROS) scavenging capacity to enhance diabetic wound healing by eliminating ROS from the wound milieu and preserving the redox balance [160]. Cao et al. developed a MoS_2_-loaded Ag ion nanosheet for the treatment of infected wounds, which demonstrated superior antibacterial efficacy compared to an equivalent quantity of silver nitrate [161]. Moreover, mesoporous silica nanospheres and bioactive glass stimulated the proliferation of fibroblasts and endothelial cells while enhancing the expression of angiogenesis-related genes through the release of silica ions [162,163]. Nanomaterials can serve as carriers for the delivery of therapeutic drugs to enhance wound healing, owing to their high specific surface area characteristics. Shi et al. created a reactive oxygen species-responsive nanoplatform encapsulating the bioactive peptide P311 to enhance diabetic wound healing. P311 exerts a therapeutic effect on granulation tissue development and re-epithelialization, while the polymeric micelles react to the oxidizing wound milieu, releasing P311 and enhancing its bioavailability [164]. Xia et al. developed a chitosan-poly (ethylene oxide) electrospun nanofiber incorporating VEGF- and PDGF-encapsulated PLGA nanoparticles, a composite nanofiber that enhances wound healing by facilitating angiogenesis and encouraging re-epithelialization [165]. Chen et al. encapsulated antioxidant small molecules (α-lipoic acid and epigallocatechin gallate) into gold nanoparticles (Au NPs), and in vivo tests revealed that the composite nanoparticles facilitated the repair of diabetic ulcers by suppressing inflammation and promoting angiogenesis [166].

The worldwide demographic is experiencing a notable aging transition, presenting new problems for bone health management [167]. The restoration of bone abnormalities resulting from trauma, congenital disorders, and tumor excision has emerged as a significant problem in recent years [168]. Advancements in science and technology have led to the increasing application of bioengineering techniques in the treatment of skeletal deformities. Owing to their superior physicochemical features, nanoparticles are poised to play a significant role in the treatment of bone abnormalities, such as enhancing the differentiation and proliferation of osteoblasts and expediting the bone healing process. The MOF can serve as a scaffold material for bone tissue engineering to promote osteoblast adhesion, differentiation, and proliferation. The pore structure of the MOF can facilitate the loading of growth factors, medicines, or other bioactive compounds for a targeted delivery at certain times and sites to promote bone repair and regeneration [169]. Tan et al. designed and constructed a biocompatible MOF-encased nano-enzymatic composite particle (CaCO_3_@ZIF@Mo-TA) that works like multiple enzymes and lets more cartilage tissue pass through it. The nanoparticles collaboratively modulated intracellular oxidative stress levels, impeded the malignant degradation of the cartilage matrix, mitigated inflammation-induced bone erosion, and facilitated and safeguarded cartilage repair [170]. Aspartic acid, as a crucial amino acid residue in osteogenic proteins, facilitates calcium phosphate deposition and bone mineralization [171]. Zhang et al. synthesized L-Asp-Cu (II) bio-MOFs (metal–organic frameworks) that gradually release Cu^2+^, demonstrate significant biocompatibility, and possess dual capabilities in enhancing MSC differentiation and angiogenesis, positioning them as a promising candidate for vascularized bone regeneration [172]. Hydroxyapatite is the predominant biomaterial utilized in bone tissue engineering, possessing an inorganic composition akin to that of natural bone, characterized by exceptional biocompatibility and osteoinductivity [173]. Scientists led by Qin developed a special hydroxyapatite nanoplatform called PDA@Au-HA. This platform can change the immune microenvironment to help bone tissue grow back by modulating the immune system. The nanoplatform exhibits synergistic properties of excellent compatibility, reactive oxygen species scavenging, and anti-inflammatory and immunomodulatory functions to expedite the bone repair process [174]. Pitrolino et al. developed a multilayered chitosan scaffold that was filled with 70% wt% nanohydroxyapatite (nHA) to make the osteoid layer stronger. This scaffold was used to fix osteochondral defects. The scaffold facilitated the adhesion and multiplication of human mesenchymal stem cells (MSCs), thereby repairing damaged cartilage and bone while creating a conducive growth environment [175].

#### 3.3.4. Nanotechnology in Preventive Medicine

Nanotechnology’s application in preventive medicine offers unique methods for early illness intervention, health monitoring, and vaccine development, hence enhancing public health and individual health management. The distinctive physicochemical characteristics of nanoparticles facilitate the efficient detection of biomarkers and the early diagnosis and warning of illnesses. Advancements in nanopreventive technologies will fundamentally transform the healthcare model, transitioning the system from a “treatment-centered” to a “health-centered” development paradigm.

Infectious diseases are ailments caused by pathogens (e.g., bacteria, viruses, fungi, or parasites) that can be transmitted between individuals or from humans to animals [176]. Infectious diseases present significant and extensive threats to human health, socio-economic stability, and public health infrastructures. Vaccination is the most effective and economical health intervention for preventing and controlling the dissemination of viral and bacterial infectious diseases that result in significant mortality and morbidity, and it effectively prevents various diseases by eliciting a robust immune response through the administration of antigenic agents [177]. Nonetheless, numerous protective vaccines (e.g., AIDS, tuberculosis, malaria, etc.) remain challenging to develop due to the intricacy of the pathogens, their rapid mutation rates, significant immune evasion capabilities, and the complexity of immune response mechanisms, resulting in elevated mortality rates for these diseases [178]. Moreover, various diseases, including cancer, benefit from mobilization via autoimmunity, which induces a humoral or cellular immune response through the introduction of tumor-associated antigens, ultimately facilitating an immune response that recognizes and eradicates cancer cells [179]. Consequently, vaccine development must surmount further challenges, and nanocarriers exhibit significant promise in the advancement of next-generation vaccines, providing numerous advantages such as enhanced CD8+ T cell responses, an optimized adjuvant and antigen co-delivery, and the capacity to transport nucleic acids [180], thereby uniquely positioning them to address vaccination challenges and explore additional applications in cancer therapy.

AIDS, TB, and malaria represent significant worldwide public health concerns that inflict extensive and severe damage to human health, socio-economic structures, and public health systems, highlighting the imperative for prevention [181]. HIV is marked by fast antigenic mutation and significant immune evasion [182]; Mycobacterium avium subsp. conjugatum possesses a substantial genome and a sophisticated immunological response [183]; and malaria is defined by a multifaceted pathogen lifecycle comprising various stages [184]. Notwithstanding the variations in pathogens, parallels exist in the vaccine development process for many diseases, with antigen delivery being paramount to immunization [185]. Using lipid nanoparticles (LNPs) as a delivery system, Melo et al. created a new self-assembling mRNA vaccine system that is designed to protect against the extracellular structural domain of the gp120 protein on the surface of the HIV-1 virus. The study hypothesis posited that sequential germline-targeted primary immunization, succeeded by targeted immunogen guidance via the mRNA platform, might elicit and steer the early maturation of certain B cell classes and their progression into broad-spectrum neutralizing antibodies (bNAbs) [186,187].

**Figure 6:**
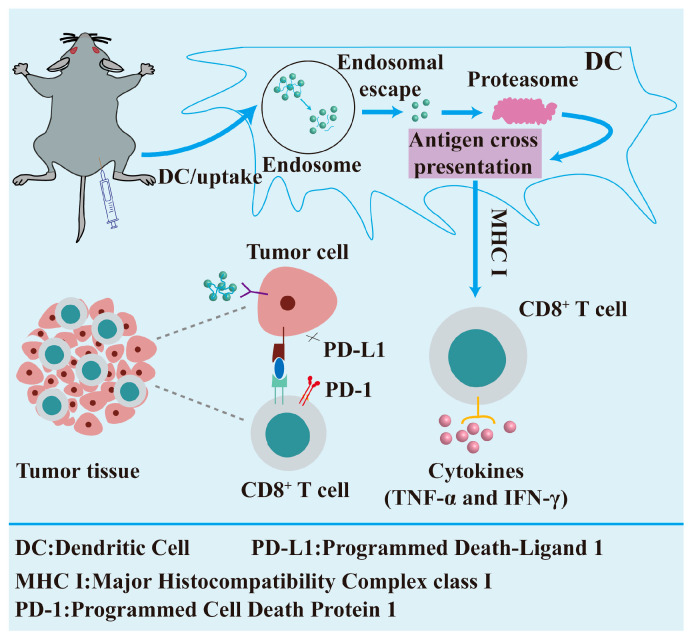
The utilization of nanotechnology in preventive medicine.

Malignant tumors represent a major hazard to human health, imposing a considerable disease burden globally. The ongoing progress in cancer vaccine research has introduced new possibilities for tailored treatment and immunotherapy. Nonetheless, certain disadvantages of cancer vaccines, like their inflexibility, inadequate immunogenicity, and enhanced specificity, have emerged throughout rigorous clinical studies [188]. Cancer nanovaccines developed by nanotechnology exhibit significant promise in tumor treatment. In contrast to conventional vaccines, nanovaccines can accurately target antigens to more suitable locations of action due to their distinctive material coupling characteristics. In order to enable antigens and adjuvants to target cells effectively, the nanoparticles’ size and the way their surfaces are modified make it easy for them to clump together in lymph nodes and the spleen, which are important immune organs. This technique boosts both the specificity of the vaccination and the robustness of the immune response [189]. The immunological receptor MR (mannose receptor) is extensively present on the cell membranes of macrophages and dendritic cells (DCs) [190]. The structure features several extracellular domains that identify and bind various endogenous and foreign ligands [191]. Researchers have designed a range of nanoparticles for vaccine delivery based on the capabilities of mRNA. Moku et al. developed a nanoparticle carrier featuring a unique sugar-based architecture. The carrier incorporated a mannoside mimic with a bis-dioxopiperazine–guanidine complex structure, and tests validated that this design markedly improved the delivery efficiency of DNA vaccines [192]. In another study (Figure 6), Chen et al. covalently conjugated mannose molecules to poly-L-lysine-riboflavin complexes (PLL-RT) to construct Man-PLL-RT nanovaccines. The Man-PLL-RT-mediated nanovaccines markedly improved the endocytosis, maturation, and cross-presentation in dendritic cells. In therapeutic applications, the combination of these nanovaccines with a PD-L1 inhibitor resulted in a notable reduction in the tumor volume in a mouse melanoma model [193].

## 4. Related Health Risks and Safety Issues

Nanotechnology’s application in the food business and health management has introduced numerous innovations and conveniences; yet, it has also generated significant worries over health risks and safety. In the food industry, while the application of nanomaterials can enhance flavor, prolong shelf life, and improve nutrient stability, their potential biotoxicity and cumulative environmental effects must not be overlooked. For instance, because of their small size, nanoscale additives (such as silver, titanium dioxide, etc.) may be able to pass through the intestinal barrier. Research has also shown that their absorption rate is three to five times higher than that of conventional materials, which may disrupt the balance of the intestinal flora and eventually cause chronic inflammation [194,195,196]. Nanomaterials in food packaging can migrate into food under specific conditions, such as elevated temperatures and acidic environments, with certain nanometallic components potentially exceeding safety thresholds [197]. Under the EU food safety framework, the Novel Food Regulation ((EU) 2015/2283) treats foods that contain engineered nanomaterials as a special case: before they can go on the market, a full safety assessment by the European Food Safety Authority (EFSA) is mandatory, and companies must supply data on the nanomaterial’s physicochemical profile (particle size distribution, specific surface area, etc.), toxicology, and stability within the food matrix [198].

The biosafety of nanoparticles is a critical concern prior to their clinical application. In health management, while nanocarriers can markedly improve nutrient bioavailability, they also heighten the risk of overdose [199]; additionally, nanomaterials in wearable monitoring devices may leach trace amounts of substances through skin contact, potentially causing adverse skin reactions [200]. Numerous nanoparticles, including silver, cadmium selenide (CdSe), zinc oxide, and iron oxide, can release metal ions that induce significant oxidative stress [201], potentially impacting various cellular signaling pathways, such as NF-κB, MAPK, Akt, and Src kinase, among others [202,203,204,205]. These pathways can induce damage to cell membranes, intracellular organelles, and nucleic acids, ultimately resulting in apoptosis or necrosis, which may trigger localized inflammation or a systemic immunological response [206].

The diverse physicochemical features of nanoparticles, including their size, surface chemistry, and form, preclude the generalization of safety and toxicity concerns [207]. The evaluation of nanoparticle toxicity necessitates a meticulous case-by-case analysis, as biological and pathological consequences are influenced by numerous variables, including the physicochemical characteristics of the nanoparticles, the exposure route, the dosage, and the length of exposure [208]. A constraint of nanomaterial risk assessments is the challenge of modeling chronic long-term exposure in laboratory animals, which possess much shorter lifespans than humans. Data regarding chronic, low-dose nanoparticle exposure may yield significant new insights into the long-term toxicological effects of nanoparticles [209]. Another limitation in the nanomaterial risk assessment is the significant variation in regulatory requirements across different regions (e.g., EU REACH regulations vs. EPA/FDA guidelines in the U.S.). Additionally, regulatory agencies worldwide have yet to establish a clear and formal set of guidelines. This uncertainty negatively impacts nanotechnology development by affecting future funding, research, and commercialization efforts. It also undermines public acceptance, potentially delaying the market introduction of nano-enabled products. Consequently, the creation of a thorough risk assessment framework and a stringent regulatory structure, along with the enhancement of scientific research on nanotechnology applications, are essential for guaranteeing their safe use in the food sector and health management.

## 5. Summary and Future Outlook

Nanotechnology is significantly revolutionizing the food business and health management via advanced materials and meticulous methods. Regarding food safety, nanomaterials can be utilized to create novel food additives and ingredients that improve the texture, flavor, color, and stability. Nanosensors facilitate the swift and highly sensitive identification of pesticide residues and pathogens, while smart packaging materials prolong shelf life, minimize waste, and provide functional foods. Nanotechnology demonstrates significant potential for application in illness diagnosis, treatment, prevention, and regenerative medicine within the realm of health interventions. Nanotechnology is utilized in the healing of human tissues, encompassing wound, bone, and corneal restoration. As a drug delivery system, it can release pharmaceuticals in response to various environmental variables, hence enhancing the therapeutic efficacy of the medications. Furthermore, nanotechnology demonstrates significant potential in the management of chronic diseases, including cancer, diabetes, and cardiovascular and cerebrovascular disorders, while concurrently advancing the production of specialized foods and tailored nutrition.

Notwithstanding the potential of nanotechnology, issues pertaining to safety, large-scale production, and regulatory control remain to be resolved. Future research endeavors should concentrate on the subsequent domains: 1. precision nutrition and individualized alimentation: the creation of sophisticated nutritional delivery systems tailored to individual metabolic requirements (e.g., nanocapsules that release micronutrients as needed); 2. The development of biodegradable nanocarriers, such as protein- and polysaccharide-based nanoparticles, to mitigate environmental and health hazards through sustainable and green nanomaterials; and 3. regulation and standardization: develop a globally unified nano-food safety assessment framework and delineate toxicological criteria and labeling requirements.

## Figures and Tables

**Table 1:** Application of inorganic nanomaterials.

Typical Shapes	Nanostructured Material	Application	References
Nanoparticle	Ag	Active packaging for food preservation and wound antimicrobials.	[8,9]
	SiO_2_	For the controlled release of pharmaceuticals, the development of very sensitive biosensors, food packaging, and seed treatments.	[10,11]
Nanowire	ZnO	Utilizing photothermal treatment for precise drug delivery and sustained release.	[12,13]
	Au	For the production of wearable high-sensitivity pressure sensors, photothermal therapy, and cardiac tissue restoration.	[14]
Nanorod	TiO_2_	For the sustained release of pharmaceuticals, cellular imaging and biomolecule tracking, and photothermal therapy.	[15,16]
Nanoplates	MoS_2_	Utilization of drug carriers; advancement of intelligent packaging materials; identification of illness markers, pathogens, and cells; and implementation of photothermal therapy.	[17,18]
Nanofilm	Cu	Advancement of intelligent packaging materials, identification of glucose, cholesterol, and other substances.	[19]
Nanoporous material	MOFs	Extended release of pharmaceuticals, including antimicrobials, oxygen scavengers, ethylene scavengers, and the detection of viral nucleic acids and antibodies.	[20]
Nanoflower	MnO_2_	Facilitating accurate drug loading and release, for food packaging, as fluorescent indicators, for wound healing.	[21]
	CaCO_3_	Utilizing drug carriers to enhance the strength, toughness, and barrier qualities of packing materials, as well as to identify biomolecules such as dopamine.	[22]

**Table 2:** Common polymer nanomaterials and their advantages.

Classification	Name of Polymer	Structure	Advantages	References
Synthetic polymer	PVA	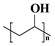	High mechanical strength and flexibility, excellent water solubility and film-forming properties, chemical modification flexibility	[31]
	PEG	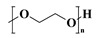	Highly hydrophilic, can be modified into amphiphilic block copolymers	[32]
	PCL	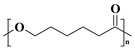	Slow degradation rate, good flexibility, low melting point	[33]
	PLGA	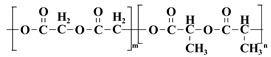	Biodegradable, high mechanical strength, but relatively brittle	[34]
	PAA	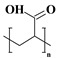	pH-responsive, highly absorbent	[35]
Natural polymer	Sodium alginate	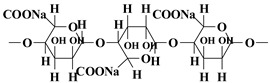	pH responsiveness, easy surface functionalization, high water absorption and water retention	[36]
	Chitosan	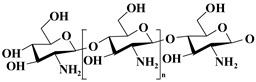	Natural antibacterial properties, good biocompatibility, and chemically modifiable	[37]
	Hyaluronic acid	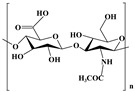	Superior water retention, targets CD44 receptor	[38]
	Sericin	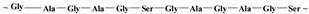	Ultra-high mechanical strength, controlled degradability	[39]
	Gelatin	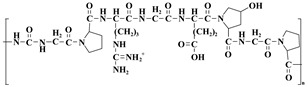	Temperature sensitivity, promotion of cell adhesion	[40]

**Table 3:** The utilization of nanotechnology in the manufacturing and processing of food.

Nanostructured Materials/Particles	Activity	Applications in Food Technology	Reference
Nanoemulsification technology	Employed to stabilize immiscible components (e.g., oil and water) and enhance the mouthfeel of sauces, dressings, and beverages.	Nanoscale emulsion droplets to improve the bioavailability of beta-carotene.	[67]
Nanopackaging technology	Encapsulates sensitive ingredients (vitamins, probiotics, antioxidants) and protects them from damage by the processing or digestive environment.	Nanoliposomes encapsulate Omega-3 fatty acids to extend shelf life and mask fish oil odor.	[68]
Nanobubble technology	Improvement of food texture and taste.	Texture optimization of low-fat ice cream.	[69,70]
Food additive	Improvement of color, taste, and stability of food, extension of shelf life.	Titanium dioxide (TiO_2_) as food additive coloring.	[71]
Nutrient enhancer	Improve the absorption efficiency of vitamins, minerals, and other nutrients.	Ascorbic acid-embedded maltolysin nanoparticles as food fortification agents.	[72]
Nanofilm	Improvement of food texture.	Nanoencapsulated nanoclusters for improved milkshake aroma.	[73]
Nano lipid rolls	Improving the quality of processed foods.		[74]
Nanocoatings	Improved abrasion resistance and antimicrobial properties of food processing equipment.	Both superhydrophobic and superhydrophilic coatings may minimize microbial adhesion to solid substrates.	[75]
Nano fertilizers/pesticides	Slow-release nanoparticles improve nutrient utilization and reduce chemical use.	Nano-copper hydroxide-controlled release pesticides.	[76]

**Table 4:** The utilization of nanotechnology in food packaging.

Nanostructured Material	Activity	Applications in Food Technology	Reference
silver-based	Demonstrates broad-spectrum efficacy against foodborne pathogens, including bacteria, fungi, yeasts, and viruses	Active packaging for food preservation	[83,84]
copper-based	UV blocking, gas barrier, mechanical, moisture sensitivity, and antimicrobial properties	Active packaging for food preservation	[85,86,87]
manganese-based	Antioxidant, enhanced barrier properties of packaging materials, environmental friendliness, intelligent sensing	For packaging of fruits and seafood, for RFID smart labels	[88,89]
zinc oxide	Antimicrobial effect, enhanced UV protection, and better barrier properties	Maintaining food quality and extending shelf life	[90,91,92]
titanium dioxide	Self-cleaning packaging surface, photocatalytic properties, stability, antimicrobial and UV-blocking capabilities, intelligent sensing	Active packaging for food preservation, inhibition of microbial growth	[93,94]
silicon dioxide	High specific surface area, chemical inertness, tunable pore structure, and good biocompatibility	Enhanced mechanical properties of packaging materials, food preservation	[95,96,97]
carbon nanomaterials	Unique mechanical, electrical, thermal, and antimicrobial properties, sustainable packaging	Antimicrobial freshness and ultra-high barrier packaging film for foodstuffs	[98,99,100]
nanoclay	Unique layered structure, high specific surface area, and modifiability	Used for fresh meat packaging, enhance the mechanical properties of packaging materials	[101]
nanocellulose	Renewable, degradable, high strength and surface area, inhibits antimicrobial effect on both Gram-negative and -positive microorganisms	Replacement of plastic laminates and super-barrier functional coatings for high-end gift packages	[102]
polymer-based	Excellent mechanical properties, renewability, crystallinity, biodegradability, and processability	Maintaining food quality and extending shelf life	[103,104]
